# Association Between the Nutritional Risk and the Survival Rate in Newly Diagnosed GIST Patients

**DOI:** 10.3389/fnut.2021.743475

**Published:** 2021-10-28

**Authors:** Ping'an Ding, Honghai Guo, Peigang Yang, Chenyu Sun, Yuan Tian, Yang Liu, Yong Li, Qun Zhao

**Affiliations:** ^1^The Third Department of Surgery, The Fourth Hospital of Hebei Medical University, Shijiazhuang, China; ^2^Internal Medicine, AMITA Health Saint Joseph Hospital Chicago, Chicago, IL, United States

**Keywords:** nutrition status, PG-SGA, NRS2002, gastrointestinal stromal tumors (GIST), risk factor, prognosis

## Abstract

**Background:** Currently, the incidence of gastrointestinal stromal tumors (GIST) is increasing rapidly worldwide. Malnutrition may increase the risk of perioperative complications and affect the prognosis of patients. However, previous studies on the nutritional status of GIST patients and its impact on prognosis are limited. Therefore, this study aims to explore the incidence of malnutrition in newly diagnosed GIST patients, the proportion of participants in need of nutritional intervention, and the relationship between nutritional status and overall survival (OS).

**Methods:** We retrospectively analyzed the clinical data of GIST patients treated in our hospital from January 2014 to January 2018. Nutritional Risk Screening 2002 (NRS2002) and Patient-Generated Subjective Global Assessment (PG-SGA) were used to assess the nutritional status of all patients. This study was to investigate the clinical significance of PG-SGA by analyzing the relationship between PG-SGA score and OS.

**Results:** A total of 1,268 newly diagnosed GIST patients were included in this study, of which 77.76% were at risk of malnutrition (NRS2002 score ≥ 3), and the incidence of malnutrition was 10.09% (PG-SGA score ≥ 4). Meanwhile, we found 2.29% of the patients required urgent nutritional support (PG-SGA score ≥ 9). Multivariate analysis showed that age (*p* = 0.013), BMI (*p* = 0.001), weight loss (*p* = 0.001), anemia (*p* = 0.005), pre-albumin (*p* = 0.010), albumin (*p* = 0.002), tumor location (*p* = 0.001), tumor size (*p* = 0.002), and NIH classification (*p* = 0.001) were risk factors for nutritional status. The prognosis was significantly in GIST patients with different PG-SGA score at admission (*p* < 0.05).

**Conclusion:** This study suggested that malnutrition is common in newly diagnosed GIST patients, and the higher the PG-SGA score, the worse the clinical outcome.

## Introduction

Gastrointestinal stromal tumors (GIST) is the most common mesenchymal tumor in the gastrointestinal tract, which is often caused by the mutation of KIT and PDGFRA genes (1, 2). The incidence of GIST is increasing at an alarming rate worldwide (3). Numerous studies have demonstrated that nutritional deficiency account for about 85% of all patients diagnosed with cancer, of whom about 50–90% have weight loss and malnutrition at the beginning of treatment (4–6). Malnutrition in patients with malignant tumors can lead to low immune function, increase perioperative infection rates, prolong hospitalization time, increase medical costs, and more importantly, affect quality of life and prognosis (7–9). In addition, the relative risk of cancer deaths caused by malnutrition is 1.8 times that of patients without malnutrition (10, 11). Therefore, it is of great importance to assess the nutritional status of patients during cancer treatment to comprehensively evaluate their tolerance to treatment.

The incidence of malnutrition in gastrointestinal tumors, especially upper gastrointestinal tumors, is higher than that in non-gastrointestinal tumors (12, 13). However, most studies investigating the gastrointestinal tumors focus on the nutritional status of non-GIST cancers, such as gastric cancer and colon cancer, while few people pay attention to the nutritional status of GIST patients, and there is no consensus on nutritional guidelines for GIST patients. As the symptoms of GIST are usually non-specific, most patients are in advanced stage at the time of diagnosis. The oppression or obstruction caused by the tumor leads to malabsorption. This results in higher risk of malnutrition, especially in patients undergoing perioperative treatment and patients with advanced and recurrent metastasis (14, 15). Therefore, correction of the malnutrition in GIST patients is particularly important for improving the quality of life and significantly prolonging the survival period.

Currently, the nutritional assessment for patients with malignant tumors generally adopts anthropometric measurements, serum biochemical indicators, Nutritional Risk Screening 2002 (NRS2002), and Patient-Generated Subjective Global Assessment (PG-SGA). According to the expert consensus of the European Society of Parenteral and Enteral Nutrition (ESPEN), once patients are diagnosed with malignant tumors, NRS2002 is the first choice for screening and assessment of nutritional status, and PG-SGA is the preferred tool for assessment (16, 17). In addition, PG-SGA was also accepted as a nutritional assessment standard for cancer patients by the Oncology Nutritional Dietetic Practice Group of the American Diet Association (18).

However, there is no standard nutritional assessment method for newly diagnosed GIST patients. And there is no conclusion the preferred choice of assessment tool for nutritional status in GIST patients. Moreover, studies on the nutritional assessment of GIST patients are scarce, with only very few studies on the application of NRS2002 combined with PG-SGA in the evaluation of newly diagnosed GIST patients. Therefore, the purpose of this study is to use the NRS2002 combined with PS-SGA score to evaluate the nutritional status of newly diagnosed GIST patients and analyze the impact of their nutritional status on prognosis.

## Methods and Materials

### Patient Section

This study retrospectively analyzed the medical data of 1,268 patients with newly diagnosed GIST admitted to the Fourth Hospital of Hebei Medical University from January 2014 to January 2018. Inclusion criteria were as follows: (1) pathological diagnosis was GIST; (2) without preoperative antitumor treatment; and (3) complete follow-up and clinical data. Exclusion criteria were as follows: (1) the patient had accepted antitumor therapies before surgery; (2) patients with cognitive impairment or other acute psychological problems; (3) without complete medical records and laboratory results; (4) the patient lost post-operative follow-up. This study was tested and approved by the ethics committee of The Fourth Hospital of Hebei Medical University, and the patients provided informed consent.

### Assessment Method

All patients were screened by NRS2002 score after admission. The score ≥3 indicates a risk of malnutrition in patients, which needs further assessment by PG-SGA. PG-SGA score includes patient self-assessment and medical staff assessment, which includes seven areas. Patients' self-assessment includes weight changes, dietary intake, self-reported symptoms, activities and function, and medical staff assessment includes nutrition-related disease status, metabolic status, physical examination. Each of these seven areas is given a score of 0–4, and the sum of scores obtained in each area is divided into quantitative and qualitative evaluations, thus providing guidance on the level of nutrition and drug intervention required by each patient. Quantitative evaluation is defined as follows: PG-SGA score of 0–1 indicates that nutritional support not required and treatment in the future based on routine re-evaluation, 2–3 points indicate malnutrition or suspected malnutrition, 4–8 points indicate moderate malnutrition, and ≥9 points indicate severe malnutrition. Qualitative evaluation indicates that patients with score of 4–8 need nutritional intervention and symptomatic treatment, and patients with score ≥9 need urgent symptomatic treatment and appropriate nutritional support before anti-tumor treatment.

### Clinicopathological Parameters and Definitions

We collected the basic data of newly diagnosed GIST patients including gender, age, weight, etc. Laboratory tests include routine blood tests and biochemical tests. Preoperative examination included abdominal computed tomography (CT), nuclear magnetic resonance imaging (MRI) and gastrointestinal endoscopy. Pathology and gene detection included tumor location, tumor size, mitotic count, immunohistochemistry, risk classification, c-kit exons 9, 11, 13 and 17, and PDGFRA exons 12 and 18. The risk classification standard we adopted is the 2008 version of the improved National Institutes of Health (NIH) classification (19).

### Follow-Up

All patients were recommended to have a follow-up visit every 3 months in the first 2 years, and every 6 months after 2 years. All patients were followed up as outpatients. The latest follow-up date was in December 2020, and the median follow-up time was 68.6 months (range 16–76 months). The total survival time was calculated from diagnosis to death or the last follow-up. Overall survival (OS) was defined as time interval from operation to tumor-related death or last contact, and OS was the preferred destination.

### Statistical Analyses

We provided PG-SGA standard questionnaires for all newly diagnosed GIST patients admitted to the Third Department of the Fourth Hospital of Hebei Medical University. Software of SPSS version 21.0 and GraphPad Prism 5.01 were utilized to perform statistical analyses. Anthropometric measurement and PG-SGA scores were expressed in the form of descriptive statistics (mean, standard deviation, and frequency), respectively. The *t* test, ANOVA test, and correlation analysis were used to statistically evaluate the degree of correlation between these factors and the PG-SGA score. Survival analysis was performed using the Kaplan-Meier method. Univariate and multivariate analyses were investigated by the Cox proportional hazards regression model. The hazard ratio (HR) and 95% confidence interval (CI) were used to assess relative risks. *P* value < 0.05 was regarded as statistical difference significantly.

## Results

### Clinicopathological Features of Newly Diagnosed GIST Patients

A total of 1,268 patients were admitted from January 2014 to January 2018, including 887 cases (69.95%) of tumors located in the stomach, 54 cases (4.26%) in the duodenum, 235 cases (18.53%) in the intestine, 30 cases (2.37%) in the colon, and 62 cases (4.89%) in the mesentery. All GIST patients were confirmed by pathology. There were 665 males (52.44%) and 603 females (47.56%), with a median age of 59 (19–76) years old. The median diameter of the tumor was 6 (2.3–15.4) cm, and the median nuclear mitotic figure was 5 (3–13) / 50HPF (Table 1).

**Table 1:** General and tumor characteristics of study participants (*n* = 1268).

**Variables**	***N* (Percentage)**
Age (years)	59.9 ± 4.2 ^*^
Sex (male)	665 (52.44%)
Weight loss	
No WL (0–1.9% of body weight)	801 (63.17%)
Mild WL (2–2.9% in 1-month or 2–5.9% in 6 months)	208 (16.40%)
Moderate WL (3–4.9% in 1-month or 6–9.9% in 6 months)	117 (9.23%)
Severe WL (5–9.9% in 1-month or10–19.9% in 6 months)	88 (6.94%)
Very severe WL (>10% in 1-month or >20% in 6 months)	54 (4.26%)
Tumor location	
Stomach	887 (69.95%)
Duodenum	54 (4.26%)
Intestine	235 (18.53%)
Colon	30 (2.37%)
Mesentery	62 (4.89%)
Tumor size (cm)	
<5.0	383 (30.21%)
5.0~10.0	789 (62.22%)
>10.0	96 (7.57%)
Nuclear mitotic figure (50HPF)	
<5	372 (29.34%)
6~10	708 (55.84%)
>10	188 (14.83%)
NIH classification	
High risk	279 (22.00%)
Moderate risk	543 (42.82%)
Low risk	309 (24.37%)
Very low risk	137 (10.80%)
Ki-67 percentage	
≤ 10%	848 (66.88%)
>10%	420 (33.12%)
c-kit exons	
Positive	961 (75.79%)
Negative	307 (24.21%)
PDGFRA exons	
Positive	319 (25.16%)
Negative	949 (74.84%)

* *Mean ± SD*.

### Nutritional Status of Newly Diagnosed GIST Patients

**Figure 1:**
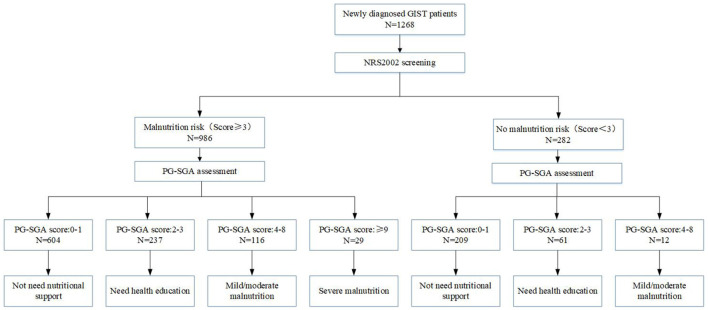
Nutrition screening and assessment of 1,268 newly diagnosed GIST patients.

Figure 1 presents the nutritional risk and assessment of 1,268 newly diagnosed GIST patients. A total of 1,268 newly diagnosed GIST patients were screened by NRS2002, and 986 patients (77.76%) had the risk of malnutrition (NRS2002 score ≥ 3), while 282 patients (22.24%) did not have the risk of malnutrition (NRS2002 score <3). The PG-SGA evaluation of 1,268 patients showed that 64.11% of the patients scored 0–1 points, indicating a good nutritional status, and nutritional support was not needed. 23.50% of the patients scored 2–3 points, so only nutritional guidance was needed. And 10.09% of the patients scored 4–8 points, indicating that there was mild/moderate malnutrition, and nutritional intervention and treatment were needed. 2.29% patients scored >9 points, indicating severe malnutrition and urgent need for symptomatic treatment and adequate nutritional support. This study also found that only 117 (74.52%) of the 157 patients who needed nutritional intervention (PG-SGA score ≥ 4) received nutritional support one week before the treatment. 2.37% of patients received parenteral nutrition (PN) support, 12.07% received enteral nutrition (EN) support, and 2.13% received both EN and PN support. In addition, we also found that 93 patients (7.33%) with good nutrition (PG-SGA score <4) received nutritional support treatment (Table 2).

**Table 2:** Patient-generated subjective global assessment classification and nutritional therapy situation (*N* = 1,268).

**Nutrition support**	**Total (%)**	**PG-SGA**			
		**0~1(%)**	**2~3(%)**	**4~8(%)**	**≧9(%)**
No	1058 (83.43)	779 (95.82)	239 (80.20)	38 (29.69)	2 (6.90)
Yes					
PN	30 (2.37)	0 (0)	5 (1.68)	14 (10.94)	11 (37.93)
EN	153 (12.07)	34 (4.18)	52 (17.45)	57 (44.53)	10 (34.48)
EN and PN	27 (2.13)	0 (0)	2 (0.67)	19 (14.84)	6 (20.69)

*PN, parenteral nutrition; EN, enteral nutrition; PG-SGA, patient-generated subjective global assessment*.

According to 2008 version NIH stromal tumor risk classification standard, 1,268 patients were divided into four groups. There were 137 patients in the extremely low risk group, 7 (5.11%) of whom were at risk of malnutrition. There were 309 cases in the low-risk group, of which 40 (12.94%) patients had the risk of malnutrition. There were 543 patients in the moderate-risk group, 220 (40.52%) of whom were at risk of malnutrition. There were 279 cases in the high-risk group, of which 188 (67.38%) patients had the risk of malnutrition. The comparison between groups showed that the risk of malnutrition in the high-risk group was significantly higher than that in the other three groups (*p* < 0.05) (Table 3).

**Table 3:** The relationship between risk classification and incidence of nutritional risk in newly diagnosed GIST patients (*N* = 1,268) [n(%)].

**Group**	***N***	**PG-SGA**				**Malnutrition incidence**
		**0~1(%)**	**2~3(%)**	**4~8(%)**	**≥9(%)**	
High risk	279	91 (32.62)	99 (35.48)	65 (12.54)	24 (8.60)	188 (67.38)
Moderate risk	543	323 (59.48)	159 (29.28)	56 (10.31)	5 (0.92)	220 (40.52)^*^
Low risk	309	269 (87.06)	33 (10.68)	7 (2.27)	0 (0)	40 (12.94)^*^
Very low risk	137	130 (94.89)	7 (5.11)	0 (0)	0 (0)	7 (5.11)^*^

* *Compared with high risk group, two-sided chi-square test, all p < 0.05*.

A total of 1,268 patients were divided into five groups according to the location of tumor. Among the 887 cases of gastric stromal tumors, 282 cases (46.61%) had malnutrition risk, while 96 of the 235 patients with intestinal stromal tumors were at risk of malnutrition (40.85%). There were 54 patients with duodenal tumors and 30 patients with colonic tumors, with 25 cases (46.30%) and 10 cases (33.33%) at risk of malnutrition, respectively. Meanwhile, there were 62 cases of stromal tumors in mesentery, and 42 cases (67.74%) had malnutrition risk. The comparison among groups showed that the risk of malnutrition in patients with mesentery stromal tumors was significantly higher (*p* < 0.05) (Table 4).

**Table 4:** Location of gastrointestinal stromal tumors and incidence of nutritional risk (*N* = 1,268) [n(%)].

**Group**	***N***	**PG-SGA**				**Malnutrition incidence**
		**0~1(%)**	**2~3(%)**	**4~8(%)**	**≥9(%)**	
Stomach	887	605 (68.21)	180 (20.29)	88 (9.92)	14 (1.58)	282 (46.61)^*^
Duodenum	54	29 (53.70)	19 (35.19)	5 (9.26)	1 (1.85)	25 (46.30)^*^
Intestine	235	139 (59.15)	73 (31.06)	17 (7.23)	6 (2.55)	96 (40.85)^*^
Colon	30	20 (66.67)	6 (20.00)	3 (10.00)	1 (3.33)	10 (33.33)^*^
Mesentery	62	20 (32.26)	20 (32.26)	15 (24.19)	7 (11.29)	42 (67.74)

* *Compared with mesentery group, two-sided chi-square test, all p < 0.05*.

### Analysis of Related Factors Affecting PG-SGA Score

Our study revealed the relationship between PG-SGA of newly diagnosed GIST patients scores and possible related factors. The PG-SGA related factors were patients' age (*p* = 0.013), BMI (*p* = 0.001), weight loss (*p* = 0.001), anemia (*p* = 0.005), pre-albumin (*p* = 0.010), albumin (*p* = 0.002), tumor location (*p* = 0.001), tumor size (*p* = 0.002), and NIH classification (*p* = 0.001) (Table 5).

**Table 5:** Analysis of PG-SGA score with factors affecting nutritional status.

**Characteristic**	**Cases (n)**	**PG-SGA score** **(Median ± SD)**	**Statistical value**	***P*-value**
Age (years)			*u* = −4.041	0.013
≥60	718	5 ± 1.66		
<60	550	3 ± 1.32		
Sex			*t* = 1.549	0.083
Male	665	4 ± 1.47		
Female	603	3 ± 1.31		
BMI (kg/m^2^)			u=11.421	0.001
<18.5	155	6 ± 2.19		
18.5~25.0	878	4 ± 1.11		
>25.0	235	3 ± 0.86		
Weight loss			*u* = 14.671	0.001
No WL (<2% of body weight)	801	3 ± 0.87		
Mild WL (2–3% in 1-month or 2–6% in 6 months)	208	4 ± 1.89		
Moderate WL (3–5% in 1-month or 6–10% in 6 months)	117	5 ± 1.36		
Severe WL (5–10% in 1-month or10–20% in 6 months)	88	6 ± 2.07		
Very severe WL (>10% in 1-month or >20% in 6 months)	54	8 ± 1.25		
Anemia			*t* = 9.997	0.005
Yes	494	5 ± 1.76		
No	774	4 ± 0.88		
Pre-albumin (mg/dL)			*t* = 23.043	0.010
<20	390	6 ± 1.34		
≥20	878	4 ± 1.11		
Albumin(g/L)			*t* = 11.034	0.002
<35	338	5 ± 1.76		
≥35	930	3 ± 0.97		
Tumor location			*u* = 10.876	0.001
stomach	887	3 ± 1.71		
duodenum	54	4 ± 1.38		
intestine	235	5 ± 1.65		
colon	30	5 ± 1.13		
mesentery	62	7 ± 1.44		
Tumor size (cm)			*u* = 13.573	0.002
<5.0	383	2 ± 1.02		
5.0~10.0	789	4 ± 0.96		
>10.0	96	7 ± 2.15		
NIH classification			*u* = 15.621	0.001
High risk	279	7 ± 2.08		
Middle risk	543	5 ± 1.17		
Low risk	309	3 ± 0.88		
Very low risk	137	3 ± 1.22		

### Treatment and Prognosis of Newly Diagnosed GIST Patients

**Figure 2:**
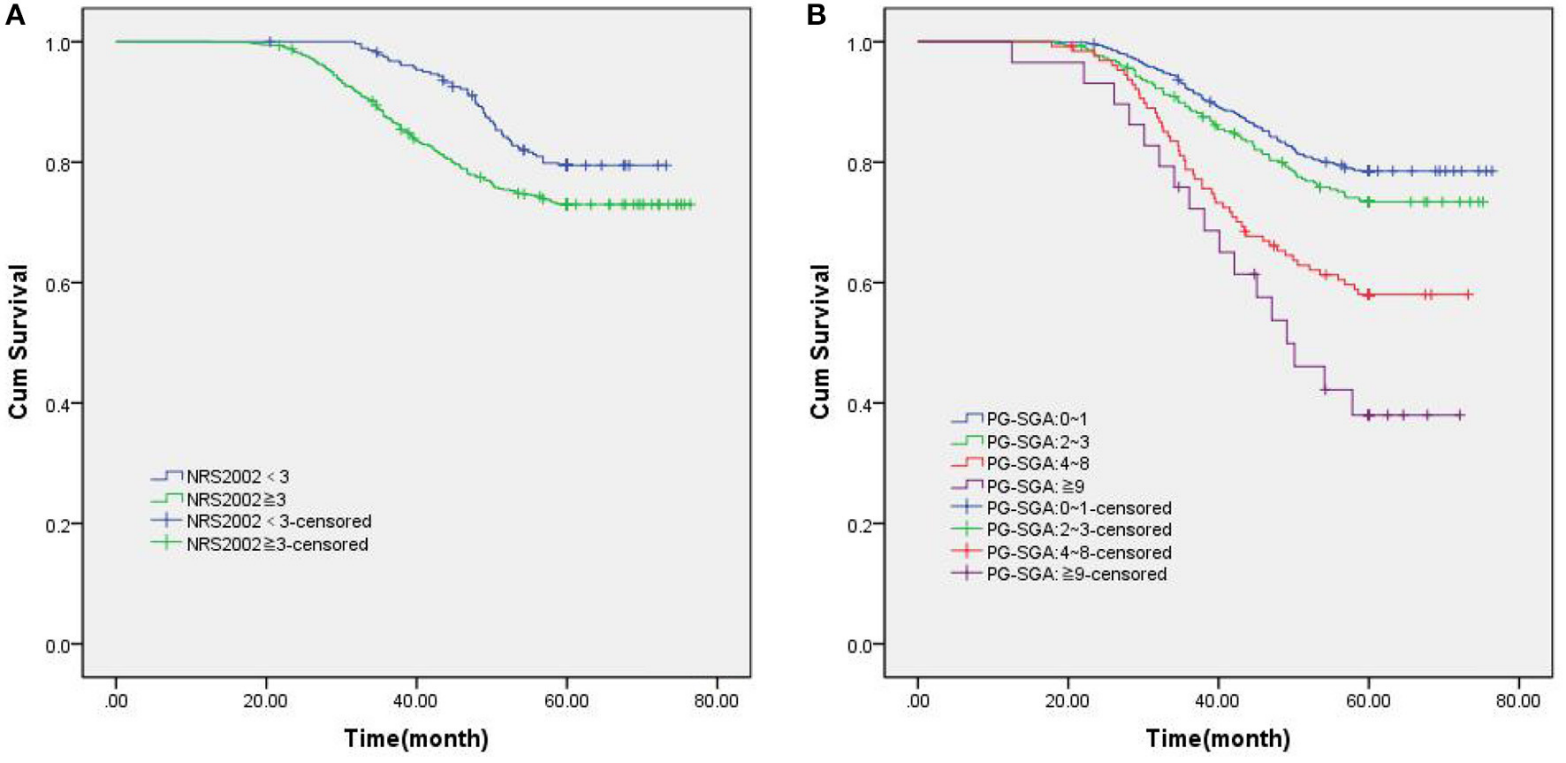
Kaplan-Meier survival curves in patients with newly diagnosed GIST patients. **(A)** Overall survival based on NRS2002 scores; **(B)** Overall survival based on PG-SGA scores.

**Figure 3:**
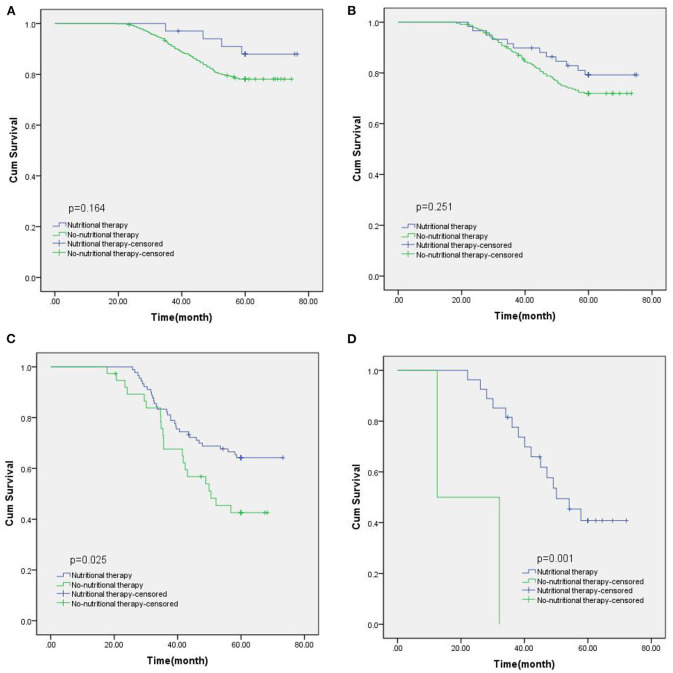
The prognosis of GIST patients with different PG-SGA scores in nutritional therapy and without any intervention. **(A)** PG-SGA scores: 0~1; **(B)** PG-SGA scores: 2~3; (**C)** PG-SGA scores: 4~8; **(D)** PG-SGA scores: ≥9.

Among 1,268 newly diagnosed GIST patients, 1,046 patients (82.49%) underwent direct surgical resection, and 222 patients (17.50%) received imatinib targeted therapy due to large tumor size. All patients were followed up for a median of 68.6 months (range 16–86). The 5-year OS rate was 74.61%, and the median survival time was 42.7 months (range 13–74). The 5-year OS rate of patients with NRS2002 score <3 was 79.79%, while that of patients with NRS2002 score ≥ 3 was only 73.12%(*p* = 0.007, Figure 2A). According to different PG-SGA scores, the stratified analysis of 1,268 patients with PG-SGA evaluation showed that the 5-year OS of patients with PG-SGA score of 0–1 and 2–3 was 78.60 and 73.93%, respectively, which was significantly better than that of patients with PG-SGA score > 3 (*p* < 0.001, Figure 2B). The prognosis of GIST patients with different PG-SGA scores in nutritional therapy and without any intervention was shown in Figure 3. For patients with PG-SGA score <4, there was no significant difference in prognosis between nutritional therapy and non-nutritional intervention (*p* = 0.164, 0.251). However, for patients with PG-SGA score of 4 ~ 8, especially those with PG-SGA score ≥ 9, nutritional therapy significantly improved the prognosis of patients, and the survival time was better than that of patients without nutritional intervention (*p* = 0.025, 0.001).

Univariate and multivariate analysis showed that PG-SGA score (*p* = 0.001, HR = 1.638, 95%CI: 1.259–2.441), pre-albumin (*p* = 0.033, HR = 0.687, 95%CI: 0.548–0.861), BMI(*p* = 0.011, HR = 1.321, 95%CI: 0.925–1.874), NIH classification (*p* = 0.000, HR = 2.805, 95%CI: 2.241–3.510), nutritional therapy(*p* = 0.012, HR = 1.267, 95%CI: 0.987–1.762) were independent risk factors affecting the 5-year OS rate of newly diagnosed GIST patients (Table 6).

**Table 6:** Univariate and multivariate analyses for OS in patients with newly diagnosed GIST.

**Variables**	**Univariate analysis**			**Multivariate analysis**		
	***P* value**	**HR**	**95% CI**	***P* value**	**HR**	**95% CI**
Gender (female vs. male)	0.010	1.321	0.898–1.624	0.079	0.758	0.556–1.003
Age (≤ 60 vs. >60)	0.065	1.457	1.123–2.083	-	-	-
NRS2002(<3 vs. ≥3)	0.033	1.593	1.256–2.763	0.066	0.895	0.431–1.346
PG-SGA (0–1 vs. 2–3 vs. 4–8 vs. ≥9)	0.008	1.832	1.360–3.321	0.001	1.638	1.259–2.441
Anemia (Yes vs. no)	0.077	0.653	0.489–1.032	-	-	-
Pre-albumin (mg/dL) (<20 vs. ≥20)	0.048	1.032	0.783–1.439	0.033	0.687	0.548–0.861
Albumin (g/L) (<35 vs. ≥35)	0.031	1.329	1.102–1.876	0.054	0.772	0.542–1.302
BMI(<18.5 vs. ≥18.5 and <25.0 vs. ≥25.0)	0.022	1.487	1.212–2.034	0.011	1.321	0.925–1.874
NIH classification (High risk vs. moderate risk vs. low risk vs. very low risk)	0.001	3.458	2.198–5.329	0.000	2.805	2.241–3.510
Nutritional therapy (No vs. yes)	0.032	1.432	1.031–1.987	0.012	1.267	0.987–1.762

## Discussion

GIST is the most common mesenchymal tumor in the gastrointestinal tract, accounting for about 2% of gastrointestinal tumors, which can occur throughout the gastrointestinal tract, also can occur in the mesangium, pelvis and retroperitoneum (20, 21). Due to the lack of specific manifestations of GIST, clinical symptoms such as gastrointestinal bleeding, abdominal pain and discomfort or abdominal mass often occur (22). The best time for early treatment has been delayed after symptom deterioration, and the 5-year survival rate has been greatly affected. Meanwhile, cancer patients are often accompanied by malnutrition in the initial diagnosis, especially digestive tract malignant tumors (23).

Our retrospective study was the first to investigate the nutritional status of newly diagnosed GIST patients and possible factors leading to malnutrition by NRS2002 combined with PG-SGA score. We found that 12.38% of newly diagnosed GIST patients were malnourished, and 2.29% of them needed urgent management to relieve malnutritional symptom and/or nutritional support. This is similar to the results of previous study conducted by Guo et al. (24) that the incidence of malnutrition was 12%, and the risk of malnutrition was 34%. Our results also discovered that malnutrition was common in newly diagnosed GIST patients, and these patients would need prompt nutrition and dietician education and guidance. This may be due to recurrent gastrointestinal symptoms such as anorexia and anorexia in GIST patients, which further leads to weight loss. In addition, the rapid growth of tumors, abdominal recurrence and systemic metastasis increased nutrition consumption, resulting in malnutrition in patients. Moreover, we found that the risk of malnutrition in patients in the high-risk group and in patients with tumors located in the mesentery was significantly higher than that in other groups.

We further analyzed the factors affecting PG-SGA score and found that tumor location and tumor risk classification were independent risk factors. Furthermore, we found that the PG-SGA score was closely related to the OS of patients. The 5-year OS rates of patients with 0–1 score were better than those of other groups, especially those with ≥4 scores. The higher PG-SGA score was associated with worse prognosis. These findings have also been supported by other studies. Ge et al. found that the nutritional status of patients with gastrointestinal cancer determines the quality of life during subsequent treatment (18). In addition, Tan et al. found that nutritional status assessed by PG-SGA may be a predictor of prognosis in patients with advanced cancer, especially in patients with gastrointestinal tumors (25). In view of these results, we speculate that PG-SGA score may exert a more effective value in evaluating the prognosis of newly diagnosed GIST patients.

There are still some limitations in our research. First, this study was a single-center retrospective study with limited number of cases. Second, only OS was investigated due to the lack of data on progression-free survival and quality of life. Therefore, further multicenter, prospective studies to more comprehensively assess the prediction values of NRS2002 combined with PG-SGA score on risk of adverse clinical outcomes in patients with GIST are still needed.

## Conclusion

In this study, NRS2002 combined with PG-SGA score was used to evaluate the nutritional status of newly diagnosed GIST patients in China for the first time to the best of our knowledge. About 12.38% of GIST patients had malnutrition at the time of diagnosis, and more than 1/10 of GIST patients needed urgent nutritional intervention and management. More attention should be paid to the nutritional status of GIST patients, especially those with high risk of malnutrition, such as elderly patients and tumors located in the mesenteric. These high-risk patients should be timely PG-SGA assessment, and give nutritional education and necessary nutritional support.

## Data Availability Statement

The raw data supporting the conclusions of this article will be made available by the authors, without undue reservation.

## Ethics Statement

This study was tested and approved by the Ethics Committee of The Fourth Hospital of Hebei Medical University, and the patients provided informed consent. The patients/participants provided their written informed consent to participate in this study.

## Author Contributions

QZ: conception and design, and administrative support. PD, PY, YT, HG, and YLi: provision of study materials or patients. PD, PY, YT, and HG: collection and assembly of data. PD and CS: data analysis and interpretation. PD, HG, PY, CS, YT, YLiu, YLi, and QZ: manuscript writing. All authors contributed to the article and approved the submitted version.

## Funding

This work was supported by the Cultivating Outstanding Talents Project of Hebei Provincial Government Fund (No. 2019012); Hebei public health committee county-level public hospitals suitable health technology promotion and storage project (No. 2019024); Hebei Medical University Education and Teaching Research Project (Nos. 2020CGPY-12, 2020CHYB-23); Hebei University Science and Technology Research Project (No. ZD2019139).

## Conflict of Interest

The authors declare that the research was conducted in the absence of any commercial or financial relationships that could be construed as a potential conflict of interest.

## Publisher's Note

All claims expressed in this article are solely those of the authors and do not necessarily represent those of their affiliated organizations, or those of the publisher, the editors and the reviewers. Any product that may be evaluated in this article, or claim that may be made by its manufacturer, is not guaranteed or endorsed by the publisher.
